# PubChem3D: Similar conformers

**DOI:** 10.1186/1758-2946-3-13

**Published:** 2011-05-09

**Authors:** Evan E Bolton, Sunghwan Kim, Stephen H Bryant

**Affiliations:** 1 National Center for Biotechnology Information, National Library of Medicine, National Institutes of Health, Department of Health and Human Services, 8600 Rockville Pike, Bethesda, MD 20894, USA

## Abstract

**Background:**

PubChem is a free and open public resource for the biological activities of small molecules. With many tens of millions of both chemical structures and biological test results, PubChem is a sizeable system with an uneven degree of available information. Some chemical structures in PubChem include a great deal of biological annotation, while others have little to none. To help users, PubChem pre-computes "neighboring" relationships to relate similar chemical structures, which may have similar biological function. In this work, we introduce a "Similar Conformers" neighboring relationship to identify compounds with similar 3-D shape and similar 3-D orientation of functional groups typically used to define pharmacophore features.

**Results:**

The first two diverse 3-D conformers of 26.1 million PubChem Compound records were compared to each other, using a shape Tanimoto (ST) of 0.8 or greater and a color Tanimoto (CT) of 0.5 or greater, yielding 8.16 billion conformer neighbor pairs and 6.62 billion compound neighbor pairs, with an average of 253 "Similar Conformers" compound neighbors per compound. Comparing the 3-D neighboring relationship to the corresponding 2-D neighboring relationship ("Similar Compounds") for molecules such as caffeine, aspirin, and morphine, one finds unique sets of related chemical structures, providing additional significant biological annotation. The PubChem 3-D neighboring relationship is also shown to be able to group a set of non-steroidal anti-inflammatory drugs (NSAIDs), despite limited PubChem 2-D similarity.

In a study of 4,218 chemical structures of biomedical interest, consisting of many known drugs, using more diverse conformers per compound results in more 3-D compound neighbors per compound; however, the overlap of the compound neighbor lists per conformer also increasingly resemble each other, being 38% identical at three conformers and 68% at ten conformers. Perhaps surprising is that the average count of conformer neighbors per conformer increases rather slowly as a function of diverse conformers considered, with only a 70% increase for a ten times growth in conformers per compound (a 68-fold increase in the conformer pairs considered).

Neighboring 3-D conformers on the scale performed, if implemented naively, is an intractable problem using a modest sized compute cluster. Methodology developed in this work relies on a series of filters to prevent performing 3-D superposition optimization, when it can be determined that two conformers cannot possibly be a neighbor. Most filters are based on Tanimoto equation volume constraints, avoiding incompatible conformers; however, others consider preliminary superposition between conformers using reference shapes.

**Conclusion:**

The "Similar Conformers" 3-D neighboring relationship locates similar small molecules of biological interest that may go unnoticed when using traditional 2-D chemical structure graph-based methods, making it complementary to such methodologies. The computational cost of 3-D similarity methodology on a wide scale, such as PubChem contents, is a considerable issue to overcome. Using a series of efficient filters, an effective throughput rate of more than 150,000 conformers per second per processor core was achieved, more than two orders of magnitude faster than without filtering.

## Background

PubChem [1-4] is a free and open public resource for the biological activities of small molecules. With more than 30 million unique chemical structures and 120 million biological test results, it is a sizeable system with an uneven degree of available information. Some chemical structures in PubChem have a great deal of biological annotation and literature associated, while many others (*e.g*., synthesized for high-throughput screening purposes) have little to nothing known about them other than the chemical structure. To help overcome this disparity, PubChem helps users to locate or relate data in the archive by pre-computing "neighboring" relationships. One of these, known as "Similar Compounds", associates a pair of chemical structures if they have a Tanimoto [5-7] similarity of 0.9 or greater when using the PubChem subgraph binary fingerprint [8] and Eq. (1).(1)

where *A *and *B *are the respective counts of fingerprint set bits in the compound pair and *AB *is the count of bits in common.

The "Similar Compounds" relationship is useful to relate analogues that may have similar biological activity or function and additional biological annotation; however, "Similar Compounds" is not particularly good at finding chemical structures that can adopt similar 3-D shape and similar 3-D orientation of functional groups typically used to define pharmacophore features (henceforth, these pharmacophore feature functional groups will be referred to as "pharmacophore features" or simply as "features"), which could indicate, for example, that the molecules bind to a protein in a similar fashion. It may be useful, therefore, to provide a "Similar Conformers" relationship in PubChem to help relate relevant conformers of chemical structures.

Wanting to compute a 3-D neighboring relationship with modest computational capacity on a very large scale and actually being able to do it are two very different things. For 30 million compounds, a neighboring relationship requires a minimum of 10^14 pair-wise similarity computations. The 2-D similarity of chemical structures with binary fingerprints is relatively fast, with rates of 10^6 compound pair similarities per second per processor core achievable. Computing the analogous 3-D pair-wise similarity of conformers is much slower, with rates of 10^2 to 10^3 per second per processor core (depending on the degree of accuracy versus performance tradeoffs one is willing to make), when using atom-centered Gaussians [9-12] for the shape description. This difference in 2-D versus 3-D pair-wise similarity overlap computation rate is made yet worse by another factor of 10^1 (or more), when considering that 3-D methods actually need to consider multiple diverse conformers per chemical structure, since a small molecule can typically adopt multiple distinct shapes or orientations of pharmacophore features at room temperature. This puts the comparable rate of computation of 3-D chemical structure pair-wise similarity overlap at least 10^4 to 10^5 slower than that for 2-D. This performance gap has led some to search for alternative approaches for determining 3-D similarity between small molecules.

In one such approach [13], 3-D similarity is recast to use a binary fingerprint to achieve a conformer pair-wise similarity overlap computation speed similar to that of 2-D similarity computation. This scheme determines a set of representative 3-D reference shapes, each corresponding to a binary bit in a fingerprint. When generating the fingerprint for a 3-D chemical structure conformer, a traditional 3-D shape superposition to all reference shapes is performed. If there is sufficient similarity to a reference shape, the corresponding binary bit is set. Besides the pre-computation expense to determine the reference shapes to use and to generate the 3-D fingerprint for all conformers to be searched, this method has an important drawback. Unlike 2-D binary fingerprint methods, when two 3-D chemical structure conformers are deemed to be similar by this approach, it might not be immediately obvious as to why. The reason is simple. The common binary bit values simply identify that the two conformers share a region of shape-space, without the additional requirement that they actually share a sufficient degree of shape similarity.

An attempt [14] was made to improve upon the 3-D binary fingerprint approach. In effect, the method was very similar; with a predetermination of reference shapes followed by 3-D shape fingerprint computation. Yet, there were a couple of important distinctions that, in essence, allowed the method to yield conformer superposition results much like "traditional" 3-D similarity superposition methods [10-12]. First, the alignment of the conformer to the reference shape was retained during shape fingerprint generation. Second, when a fingerprint "bit" was in common between two conformers, the retained alignments to the reference shape were used to yield an (approximate) alignment between the conformers. Dubbed "alignment recycling", this approach recognized that conformers with similar shape align to a reference shape in a similar way. By "replaying" the alignment to common reference shapes, the best superposition between the conformer pair is the result of the similarity computation. This approach, while not as fast as the method that used only a binary fingerprint, was 10^2 times faster than "traditional" 3-D similarity superposition methodology. A major downside to "alignment recycling" was that it was only parameterized for relatively small and inflexible chemical structures. It means that additional work is necessary to extend this approach to larger and more flexible structures. In all, the above two 3-D fingerprint approaches showed great promise to dramatically improve the throughput of 3-D similarity computation.

To harness a 3-D fingerprint to speed 3-D similarity throughput, one must first determine the reference shapes to use. Recent efforts [13] to describe the shape space of biologically relevant small molecules showed exponential behavior in reference shape count resulting from changes to the minimum shape Tanimoto (ST) distance between reference shapes. However, when examining the growth of shape space per unit volume for a maximum count of reference shapes [15], shape space was shown to grow gradually and smoothly as a function of ST. In addition, and generally speaking, it was shown that the shape space of a given unit volume describes 40-70% of the shape space of all chemical structures with a lesser volume. This would suggest that one could group together regions of shape space and describe it with a relatively small number of reference shapes, while avoiding the problem of having too many reference shapes. Reformulating the fingerprint definition with multiple tiers of fingerprints with different minimum ST distances between reference shapes may allow "alignment recycling" to be effective for larger and more flexible chemical structures, thus, providing a means to speed computation of a 3-D neighboring relationship on a very large scale.

In this work, we describe the multi-conformer PubChem "Similar Conformers" 3-D neighboring relationship and explain various strategies and approaches that made it a tractable problem, including extending the "alignment recycling" methodology to cover the full range of chemical structures considered in the PubChem3D project.

## Results and discussion

### 1. Description of "Similar Conformers" neighboring relationship

PubChem uses two 3-D similarity measures to determine whether two molecules are "Similar Conformers". One of these is the shape Tanimoto (ST) for shape similarity [10-12,16,17], given by Eq. (2). The second similarity measure, defined by Eq. (3), is the color Tanimoto (CT) [10,12], which quantifies the 3-D shape similarity of fictitious "color" atoms, each representing the 3-D location of a particular pharmacophore feature functional group type: hydrogen-bond donor, hydrogen-bond acceptor, cation, anion, hydrophobe, or ring. The ST and CT values range between 0 (for no similarity) and 1 (for identical).(2)

where *V_AA _*and *V_BB _*are the respective self-overlap volume and *V_AB _*is the overlap volume of conformers A and B.(3)

where, for each of the six independent fictitious feature atom types, *V_AA _*and *V_BB _*are the respective self-overlap volumes and *V_AB _*is the overlap volume of conformers A and B.

Pair-wise shape and feature comparison of conformers takes two basic steps: (1) optimization of the shape superposition between two 3-D chemical structures, to find their maximum shape overlap in terms of ST, and (2) a single-point CT computation at that maximum shape overlay. PubChem 3-D "Similar Conformers" neighbors are identified as any pair-wise conformer superposition with ST and CT values of ≥0.8 and ≥0.5 (actually ≥0.795 and ≥0.495, after floating point number rounding is considered), respectively.

An important issue with 3-D neighboring is the number of conformers considered. Although PubChem generates a conformer ensemble for each molecule, consisting of up to 500 sampled conformations, it is not practical to consider all of these for 3-D neighboring. Therefore, a selection of diverse conformers for each compound is considered for the purposes of 3-D neighboring. A detailed description of how the diverse conformer set is derived can be found in the **Materials and Methods **section (See "Diverse conformer concept").

It is important to note that 3-D neighboring using a single conformer per compound has a one-to-one correspondence between compound pairs and conformer pairs. When using multiple conformers per compound, it is possible that only a subset of possible conformer pairs per compound pair may satisfy the 3-D neighboring criteria. For clarification, a 3-D *conformer neighbor *pair is defined as any conformer pair with ST ≥ 0.8 and CT ≥ 0.5. If there is at least one *conformer neighbor *pair among all possible conformer pairs from a given compound pair, a *compound neighbor *pair results. In this work, a 3-D neighbor implies a 3-D *compound *neighbor. If further clarification is necessary, the terms 3-D compound neighbors and 3-D conformer neighbors are used.

### 2. The distribution of 3-D neighbors

**Figure 1:**
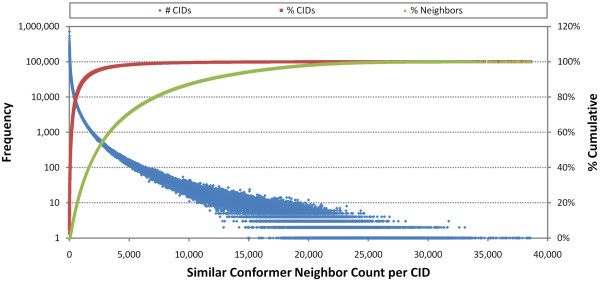
**Count of "Similar Conformers" per compound**. The frequency of unique 3-D compound neighbors counts per PubChem Compound record (CID) [blue diamond], percent cumulative CID count [red square], and percent cumulative 3-D neighbor count [green triangle].

**Figure 2:**
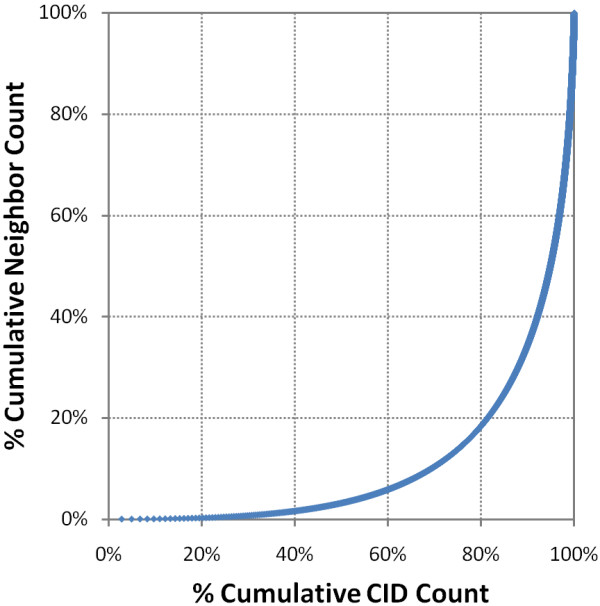
**Most compounds have few 3-D neighbors**. More than 80% of all CIDs have only 20% of 3-D neighbors.

At the time of writing, 26,153,061 PubChem Compound records (CIDs) have a "Similar Conformers" neighboring relationship using the first two diverse conformers per compound. These identified 6.62 billion unique compound neighbor pairs and 8.16 billion unique conformer neighbor pairs. The average compound neighbor count per compound, after exclusion of self-neighbor pairs, is 253. Figure 1 shows the frequency of neighbor count per compound, cumulative % CID count, and cumulative % 3-D neighbor count. Although some CIDs have more than 30,000 neighbors, 21.9 million CIDs (87.5%) have less than 1,000 neighbors, and 1.12 million CIDs (4.27%) do not even have a neighbor beyond self. This rather skewed population of the neighbor count per CID is reflected in the plot of % cumulative neighbor count versus % cumulative CID count (Figure 2). One can see that 20% of the chemical structures have more than 80% of the "Similar Conformer" neighbor pairs.

**Figure 3:**
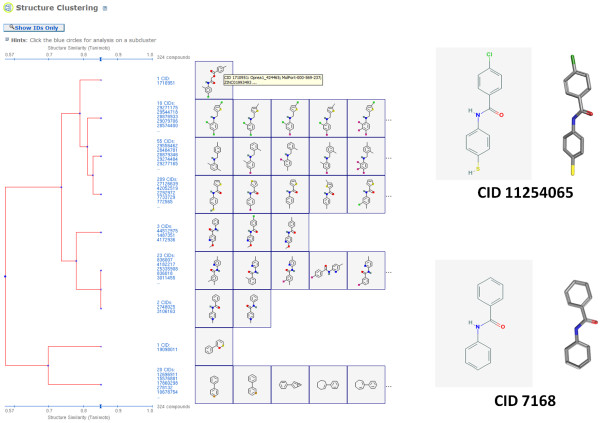
**Compounds with the most 3-D neighbors**. The PubChem Structure Clustering analysis of the 324 PubChem Compound records with more than 30,000 neighbors shows a common structural motif of two (aromatic) rings separated by a linker. N-phenylbenzamide (CID 7168) scaffold is present in the majority of these, with CID 11254065 having the most 3-D neighbors in all of PubChem.

The chemical structures on the extreme end, with more than 30,000 neighbors each, have a common motif of two substituted aromatic ring systems separated by different linkers. Figure 3 depicts a single-linkage clustering of all 324 chemical structures with more than 30,000 3-D neighbors performed with the PubChem Structure Clustering tool using the PubChem 2-D dictionary-based binary fingerprint and Eq. (1) to help highlight the different chemical series represented. The most prevalent of these are based on N-phenylbenzamide (CID 7168). Neighboring reflects the contents of PubChem. If there is a large subpopulation of chemical structures very similar to each other, those chemical structures will interrelate; however, one advantage of 3-D "Similar Conformers" neighboring is that it relates chemical structures that have similar shape and features, which can be somewhat orthogonal to a chemical series identified by 2-D "Similar Compound" neighboring (to be discussed in more detail in the next section).

**Figure 4:**
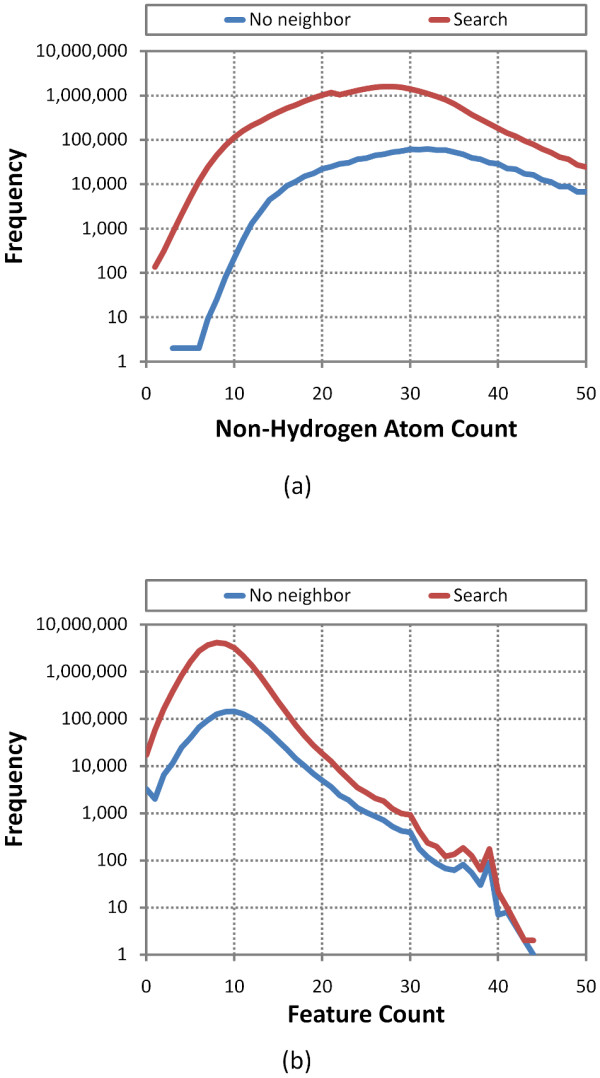
**Molecules without neighbors**. Non-hydrogen atom count and feature atom count profiles for the 1.12 million CIDs without a neighbor pair (other than the self-neighbor pair) compared to those for all 26.1 million neighbored CIDs (*Search *set), showing "no neighbor" cases are found across the entire range but accounting for much of the larger count cases.

Of the 1.12 million CIDs without a neighbor pair, except for self, these include a large and significant percentage of the total cases where the count of atoms or features is high, as depicted in Figure 4. The lack of 3-D neighbor means that these larger compounds lack a 3-D complement, which is not surprising given that shape space grows exponentially and PubChem3D limits consideration to chemical structures with fifty or fewer non-hydrogen atoms, making it increasingly less likely that a suitable neighbor can be found as a function of volume. Otherwise, the profile of chemical structures without neighbors is much like that for a set of 26,157,365 CIDs that represent the entire "live" PubChem3D contents as of October 2010 (designated as the *Search *set), representing a small minority of chemical structures with unique shape and feature profiles. For the first and second diverse conformers per compound, respectively, there are 1.31 million and 4.77 million cases where only the self neighbor is found. Employing a second diverse conformer allows 0.19 million additional CIDs to have a compound neighbor beyond self. The big increase in self-only neighbor pairs for the second diverse conformer, which represents the conformer most dissimilar to the first in a conformer ensemble, is notable; however, it is too early to say definitively whether these counts of no-neighbor per conformer will remain high, as more diverse conformers per compound are considered.

### 3. Comparison of 2-D and 3-D similarity neighbors

**Figure 5:**
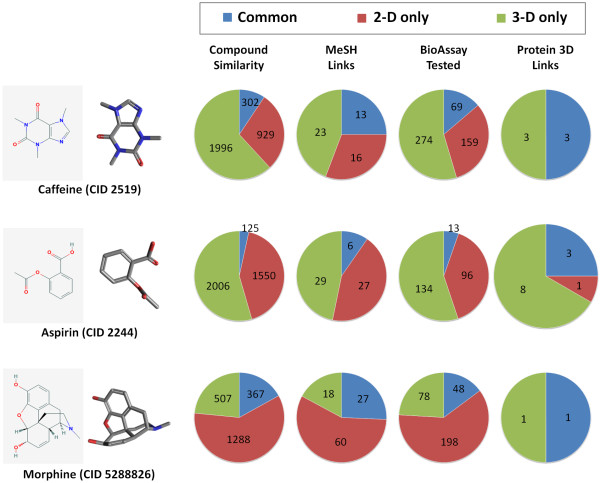
**2-D neighbors versus 3-D neighbors**. Comparison of the 2-D "Similar Compound" and 3-D "Similar Conformer" neighboring relationships using three well known small molecules, caffeine, aspirin, and morphine, demonstrates how each neighboring relationship can help find related chemical structures with unique biological annotation.

For a given molecule, PubChem provides a "Similar Compounds" 2-D neighboring relationship, computed using a 2-D binary fingerprint and a threshold of 0.9 Tanimoto similarity using Eq. (1). It is interesting to see how one can find related biological annotation information using the 3-D "Similar Conformers" neighboring relationship as opposed to the 2-D "Similar Compounds". To demonstrate this, three well known molecules of biomedical interest are selected: caffeine (CID 2519), aspirin (CID 2244), and morphine (CID 5288826). The overlap of three primary types of annotation is examined. The metrics used are unique and common count of neighbors with links to: Medical Subject Heading (MeSH) [18], through which one can locate scientific literature about a similar chemical structure in PubMed [19]; PubChem BioAssay database [3], where one can find biological and experimental data, including protein binding inhibition values; and protein 3-D structures [20], representing 3-D structures of a discrete protein with a bound ligand, determined by X-ray crystallography or NMR spectroscopy. Figure 5 gives the overlaps found between 2-D and 3-D neighboring relationships. As one can see, caffeine has 1,231 2-D neighbors, but only 302 of these are in common with its 2,298 3-D neighbors. The non-overlapping parts between the 2-D and 3-D neighboring show how similar, yet unique, chemical space is located. Of the unique 3-D neighbors, they expand, beyond its 2-D counterpart, the available biomedical annotation that may be related and relevant, with an additional 23 MeSH links, 274 biological experiments, and a doubling of the protein 3-D structures to consider. A similar result is found in the case of aspirin and morphine. It appears clear that in these cases 3-D similarity complements 2-D similarity with a mostly unique set of chemical structures that help one to discover connections between small molecules that might otherwise be missed. While this near orthogonality of neighbor sets won't be true for all chemical structures, it can be helpful to locate and relate available information in a vast data system such as PubChem.

**Figure 6:**
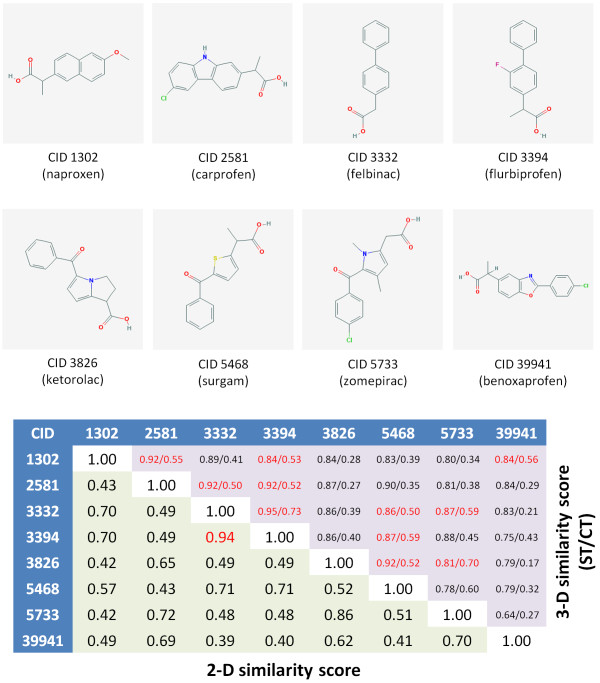
**Similarity score matrix for selected non-steroidal anti-inflammatory drugs**. The lower triangle of the score matrix corresponds to the 2-D similarity scores computed using the PubChem fingerprint, and the upper triangle corresponds to the 3-D similarity ST/CT scores. The matrix elements in red indicate the 2-D "Similar Compounds" (with a 2-D score of ≥ 0.9) or 3-D "Similar Conformers" (with a 3-D score of ST ≥ 0.8 and CT ≥ 0.5). The first ten diverse conformers were used for each molecule.

**Figure 7:**
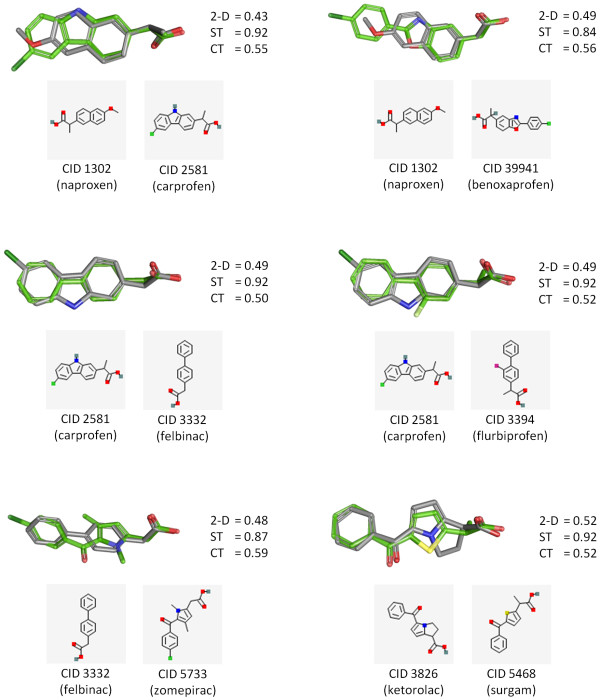
**3-D superposition of selected 3-D "Similar Conformers" pairs**. Although there is little 2-D similarity, using the PubChem fingerprint, significant 3-D similarity are found between selected non-steroidal anti-inflammatory drugs.

To further emphasize how the 3-D "Similar Conformers" neighboring relationship may complement the 2-D "Similar Compounds" neighboring relationship, the 2-D and 3-D similarity scores of eight drug molecules with the same mechanism of action are compared in Figure 6, and the 3-D alignment for particular compound pairs, whose 2-D and 3-D similarity difference are relatively large, are depicted in Figure 7. All eight drugs are known inhibitors of prostaglandin synthase [21-25] and were carefully selected for illustrative purposes from the PubChem Compound database via the MeSH pharmacological action of "anti-inflammatory agents, non-steroidal" (MeSH ID 68000894), also known as NSAIDs. While the 2-D similarity between drug molecules is calculated using the PubChem subgraph fingerprint [8], the 3-D similarity scores represent the best ST and CT similarity values from all possible combinations of the first ten diverse conformers of each compound pair. Although all eight molecules inhibit the same target, only one molecule pair (CIDs 3332 and 3394) is identified as a 2-D neighbor, as shown in the lower triangle of the similarity score matrix. The 3-D similarity approach, however, identified 11 molecule pairs as 3-D neighbors. For example, although the 2-D similarity score between CIDs 1302 and 2581 is 0.43, there are significant 3-D shape and feature overlaps (ST = 0.92 and CT = 0.55) between them (Figure 7). If fewer conformers are used, the number of resulting 3-D "Similar Conformers" neighbor pairs will be reduced. When using 2, 3, 5, 7, and 10 diverse conformers, a total of 2, 3, 9, 11, and 11 compound pairs and 2, 3, 14, 22, and 27 conformer pairs, respectively, met the 3-D neighboring criteria for the eight drug molecules.

While not all eight selected NSAID drug molecules are 3-D neighbors of each other, examining the 3-D neighbors of the 3-D neighbors shows that each of the eight drug molecules is related to one or more of the eight drug molecules, effectively forming a cluster of related drugs that are highly similar in terms of shape and pharmacophore features but rather dissimilar in terms of 2-D graph similarity. Actually, this "cluster" of NSAID drugs presented in Figure 6 is part of a larger 3-D cluster, with only eight of thirteen members being selected for clarity and demonstrative purposes. In addition, this is only one of several NSAID drug "clusters" that one can find using 3-D similarity. For the purposes of brevity and focus, only the drug class NSAIDs is explored, but suffice it to say that there are other examples one can find with other drug target classes that are similarly demonstrative.

If a molecule has known bioactivity, there is a reasonable expectation [26,27] that its similarity neighbors may also be similarly bioactive. As demonstrated in Figure 6 and 7, the 3-D "Similar Conformers" relationship can be useful to identify structurally similar molecules that may be completely missed when only the 2-D "Similar Compounds" relationship is exploited. Therefore, one might consider to use PubChem's precomputed 2-D and 3-D neighboring relationships as complementary virtual screening tools or to help understand how chemical structures relate to each other relative to their biological efficacy.

### 4. Effect of using multiple conformers

Taking into account all conformers of each CID for 3-D neighboring using the current methodology is simply not practical. The PubChem "Similar Conformers" neighboring relationship described here considers (at the time of writing) only two diverse conformers per compound (with a third conformer per compound soon to be released). One may wonder, as more conformers are considered, does one locate more chemical structures and, if so, to what extent? Is there a point of "diminishing returns", where a plateau forms in the curve of unique neighbor count as a function of diverse conformer count? Indirect evidence addressing aspects of these questions can be found in the 3-D neighboring data PubChem provides.

**Figure 8:**
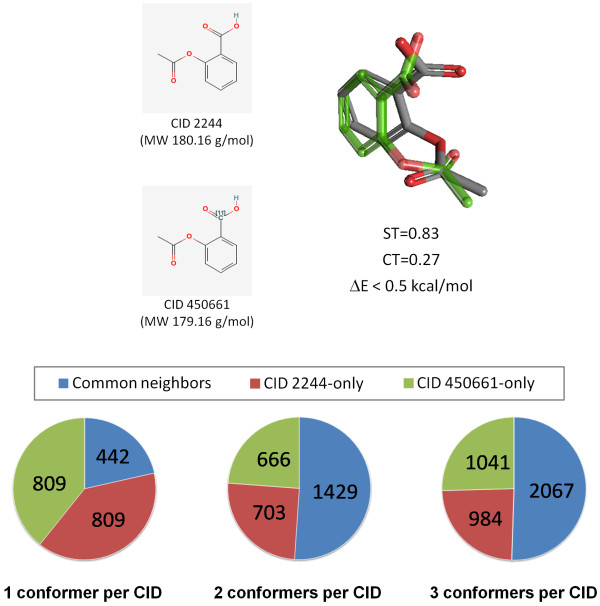
**Sensitivity of conformer choice in 3-D neighboring**. Independent conformer processing for CID 2244 and CID 450661, which differ by a single isotope, resulted in default conformers that are effectively mirror images. The 3-D neighbors are different, but less so as more diverse conformers are used, illustrating the sensitivity of the input conformers to 3-D neighboring, but also how using multiple conformers during neighboring can help mitigate such effects.

PubChem assigns different unique compound identifiers (CIDs) for different isotopomers of the same chemical structure. For example, CID 2244 and CID 450661 are both aspirin (Figure 8), but they differ from each other in the mass of one of the carbonyl carbon atoms. Although they are effectively identical for 3-D neighboring purposes, the conformer generation processing employed in PubChem3D resulted in different "default" conformers that are effectively mirror images of each other, with an insignificant energy difference of less than 0.5 kcal/mol. Superposition of the default conformers of these two CIDs yields a ST of 0.83, meeting the ST neighboring threshold; however, the CT at this superposition is only 0.27, which is not similar enough to satisfy the "Similar Conformers" 3-D neighboring threshold. As shown in Figure 8 and Table 1, the neighbors for the first three diverse conformers of CID 2244 and CID 450661 each have some degree of overlap, and, in some cases, this overlap is significant. For example, 62% (775 of 1,251) of the 3-D neighbors for the first diverse conformer of CID 2244 are identical to the 3-D neighbors found for the second diverse conformer of CID 450661. Similarly, 63% (812 out of 1,296) of the 3-D neighbors of the second diverse conformer of CID 2244 overlap with those of the first diverse conformer of CID 450661, while the third diverse conformer of CID 2244 shares 60% (730 out of 1,214) of its neighbors with the third conformer of CID 450661. Although there is a great deal of similarity between different chosen conformers of aspirin, they still identify a sizeable population of unique 3-D neighbors between CID 2244 and CID 450661, and, thus, unique shape/feature space. This demonstrates the sensitivity of the conformers used during neighboring processing, even for simple chemical structures like aspirin; however, considering PubChem is using a diverse conformer scheme, as more conformers are used in neighboring, the coverage of the conformational variation improves. This leaves one to wonder, how many diverse conformers per compound might be necessary to saturate this coverage and moderate the effects of this sensitivity?

**Table 1:** Sensitivity of conformer choice in 3-D neighboring.

	CID 2244 **1st conf**.	CID 2244 **2nd conf**.	CID 2244 **3rd conf**.	CID 450661 **1st conf**.	CID 450661 **2nd conf**.	CID 450661 **3rd conf**.
CID 2244 1st conf.	**1251 (100%)**	411 (32%)	188 (15%)	442 (35%)	775 (61%)	194 (14%)
CID 2244 2nd conf.	411 (33%)	**1296 (100%)**	186 (15%)	812 (65%)	519 (41%)	219 (16%)
CID 2244 3rd conf.	188 (15%)	186 (14%)	**1214 (100%)**	194 (16%)	219 (17%)	730 (54%)
CID 450661 1st conf.	442 (35%)	812 (63%)	194 (16%)	**1251 (100%)**	420 (33%)	229 (17%)
CID 450661 2nd conf.	775 (62%)	519 (40%)	219 (18%)	420 (34%)	**1265 (100%)**	193 (14%)
CID 450661 3rd conf.	194 (16%)	219 (17%)	730 (60%)	229 (18%)	193 (15%)	**1340 (100%)**

"Similar Conformers" 3-D neighbor overlap between the first three diverse conformers of aspirin, represented by CIDs 2244 and 450661. Numbers in parentheses are the percentage of common neighbors relative to the diagonal element in the same column.

To help address this question more directly, 4,218 compounds were 3-D neighbored against all of PubChem3D. This set of 4,218 compounds were selected using a query of the PubChem Compound database (*"has pharm"[Filter] AND "has 3d conformer"[Filter] AND 0[AtomChiralUndefCount] AND 0[BondChiralUndefCount]*). This query means that the queried chemical structures have known pharmacological action as annotated by MeSH [18], have a conformer model in PubChem3D, and have zero undefined SP2/SP3 stereo centers. (The last criterion is utilized solely to limit the count of chemical structures considered and should have no bearing on the results of this test.) The PubChem CIDs for the selected chemical structures are available in Additional file 1.

**Figure 9:**
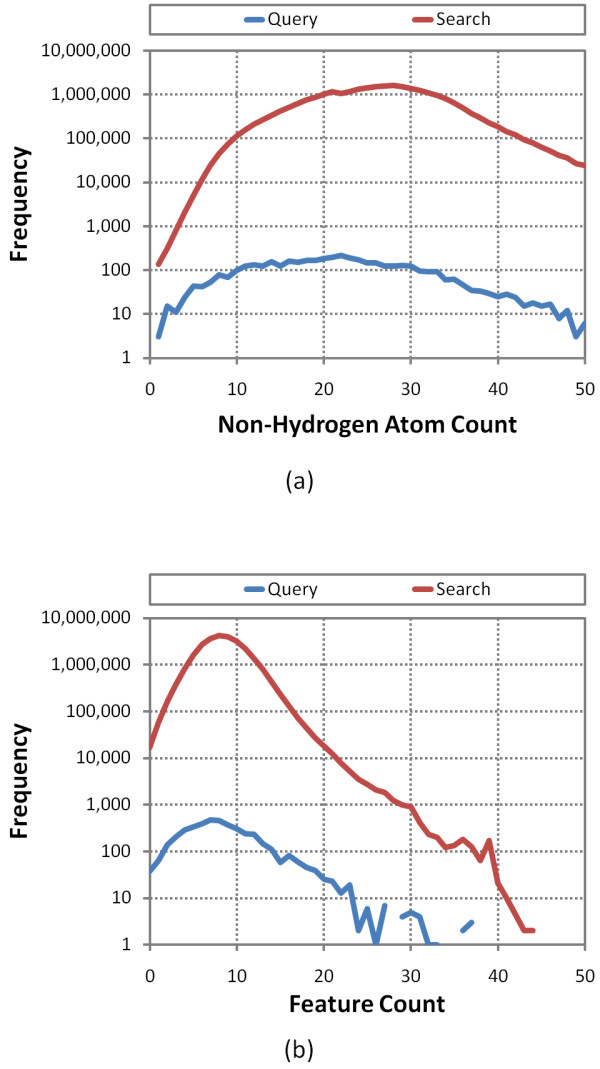
***Query *and *Search *set profile comparison**. Frequency plot of the counts of non-hydrogen atoms and features for the 4,218 chemical structures with known pharmacological action (*Query*) and all 26,157,365 PubChem3D Compound records (*Search*).

These molecules were selected as they are among the most biologically relevant small molecule chemical structures known, being heavily studied in the biomedical literature and consisting, in large part, of most known drugs. Of the very broad range of 367 pharmacological actions defined for the 4,218 small molecules, the three with greatest compound count were enzyme inhibitors (336), anti-bacterial agents (237), and antineoplastic agents (230). These small molecules with known biological action (*Query set*) were neighbored against 26,157,365 compound records (*Search set*), representing the entire "live" PubChem3D contents as of Oct. 2010, using up to 1, 3, 5, 7, and 10 diverse conformers per compound for both compound sets. As shown in Table 2, the average conformer counts between the *Query *set and *Search *set are similar, with the query set being slightly less flexible. The non-hydrogen atom count and feature count profiles depicted in Figure 9 for the *Query *set are also comparable to those found for the *Search *set.

**Table 2:** Effect of using multiple conformers per compound on 3-D neighboring.

	**Diverse Conformer Count**				
	
	**1**	**3**	**5**	**7**	**10**
	
Average *Query *Conformers per compound	1.0	2.7	4.1	5.4	7.2
Average *Search *Conformers per compound	1.0	3.0	4.9	6.7	9.4
Total Compound Pairs (billions)	110	110	110	110	110
Total Conformer Pairs (billions)	110	866	2,200	4,010	7,510
Conformer Pairs per Compound Pair	1.0	7.9	20.0	36.4	68.1
Total 3-D Compound Neighbors (millions)	1.82	3.75	5.06	6.02	7.05
Total 3-D Conformer Neighbors (millions)	1.82	6.06	10.75	15.50	22.33
Ratio of Conformer/Compound Neighbors	1.0	1.6	2.1	2.6	3.2
Average Compound 3-D Neighbors per Compound	432	889	1,200	1,428	1,673
Average Conformer 3-D Neighbors per Conformer	432	541	621	677	734
Total Search Time (days)	8.2	68.5	163.1	296.9	564.9

Growth of unique 3-D neighbors as a function of diverse conformer count for the 4,218 chemical structures (*Query*) with known biological function when neighbored against the entire "live" PubChem3D contents (*Search*) of 26,157,365 small molecules.

**Figure 10:**
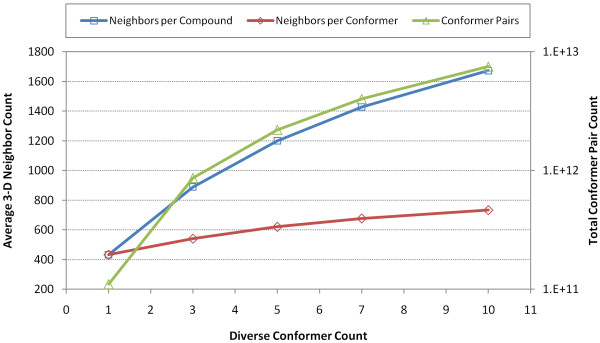
**Compound 3-D neighbor count correlated to Log(Conformer pair count)**. Plot of count of 3-D neighbors per compound and neighbors per conformer [left Y-axis] and a plot of Log_10_(total conformer pairs) [right Y-axis] as a function of diverse conformer count considered during neighboring.

Looking at Table 2, one can see that the average counts of neighbors per conformer and those per compound increase as a function of diverse conformer count. Interestingly, as shown in Figure 10, the average count of compound neighbors per compound appears highly correlated with the logarithm of total conformer pairs considered by neighboring. This suggests one must exponentially increase the count of conformer pairs to achieve a complementary linear increase in unique compound pairs.

It is not completely clear why this should be so, but one consideration comes to mind. It may be an artefact of the nature of the diverse conformer relationship, whereby a default conformer is chosen as the first, the most diverse conformer to the default conformer is the selected as the second, and each subsequent diverse conformer must be furthest away from the previously selected diverse conformers. This means that the most diverse conformers for a chemical structure are always considered first. Subsequently, each additional diverse conformer will increasingly resemble the previous diverse conformers, potentially yielding compound neighbors found previously by the other conformers for the same chemical structure. This is reflected by the ratio of conformer and compound 3-D neighbors. At three, five, and seven diverse conformers, 38%, 53%, and 61%, respectively, of the conformer neighbors point to the same compound neighbors. By ten diverse conformers, 68% of the conformer neighbors point to the same compound neighbors. With this said, one thing is clear. Neighboring more diverse conformers per compound will result in more compound neighbors per compound; however, the computation effort expended to do this grows exponentially as an increasing ratio of conformer neighbors show you more ways two compounds are interrelated.

One interesting aspect of Table 2 and Figure 10 is that the average conformer neighbor count per conformer grows very slowly. A ten times growth in conformers, corresponding to a 68 times increase in conformer pairs considered, results in only a 70% increase in the average conformer neighbor count. This is somewhat surprising given the argument above. It appears to suggest that each added diverse conformer of a chemical structure is also adding a significant portion of unique shape/feature space. This is seen in Table 1, whereby the conformer neighbors of each of the first three diverse conformers of aspirin (CID 2244 or CID 450661) mostly had very little overlap, typically less than 20%, of similar conformer neighbors with other diverse conformers of the same chemical structure. While the degree of unique shape/feature space being added may diminish as more diverse conformers are added, it would still appear to be rather substantial even at ten diverse conformers per compound. Eventually, one may expect, as even more diverse conformers are considered, that the average count of conformer neighbors per conformer may grow substantially, as conformers increasingly yield similar neighbor lists, but clearly this point is not yet reached at ten conformers per compound, as reflected by the continued growth in average count of compound neighbors per compound. Perhaps, for most chemical structures, this point may be reached by twenty diverse conformers. Using the computers and algorithms of today, and as reflected in the total search time in Table 2, twenty diverse conformers per compound is still a mountain too high to climb for a collection of the size of PubChem.

### 5. Efficiency of 3-D neighboring scheme

Although the overall speed of 3-D neighboring depends on various factors, such as atom count, use of a precomputed shape grid approach, etc., a modern computer processor core can process on the order of 10^2 to 10^3 3-D conformer pair superpositions per second, when using a Gaussian-based shape definition. In theory, 26.1 million compounds with two diverse conformers per compound would require more than a quadrillion (10^15) pair-wise conformer superposition determinations, corresponding to +40,000 years of processor core computation; however, PubChem 3-D neighboring processing was completed in about two months using ~2,500 computer processing cores (which represents more the throughput achieved in terms of actual time on a somewhat chaotic and somewhat unstable shared compute cluster rather than actual CPU time), meaning it took ~400 years of compute server time. How was this achieved?

To demonstrate the efficiency of the PubChem3D neighboring system, and reusing the previous example of querying 4,218 known bioactive small molecules against all of PubChem, Table 3 gives the percentage of conformer pairs excluded by filter type and the percentage of time spent in each stage of the neighboring processing. In the first stage, a series of three filters are utilized to screen out conformer pairs incapable of achieving the ST and CT thresholds of 0.8 and 0.5, respectively, required to be a neighbor. The most effective of these is the CT feature filter with 65% efficiency for this test set, which is to say more than half of all conformer pairs encountered can be effectively ignored. One nice aspect is that this CT feature filter operates on compound pairs, as opposed to conformer pairs. The other two filters at this stage check for incompatible shape or feature volume between conformer pairs. The total CPU time spent performing these three filters is less than 1%, yet they are effective, removing 68% of all conformer pairs from further consideration.

**Table 3:** Performance of 3-D neighboring.

	**Diverse Conformer Count**				
	
	**1**	**3**	**5**	**7**	**10**
	
Total Conformer Pairs (billions)	110	866	2,200	4,010	7,510
CT Feature Filter	65.7%	65.2%	65.0%	64.9%	64.7%
CT Volume Filter	0.2%	0.2%	0.2%	0.2%	0.2%
ST Volume Filter	2.9%	2.7%	2.6%	2.5%	2.3%
Alignment Recycling Fingerprint	4.4%	4.5%	4.5%	4.6%	4.7%
Alignment Recycling Overlap	26.5%	27.2%	27.5%	27.7%	27.9%
Insufficient ST (billions)	0.2	1.3	2.9	5.0	8.5
Insufficient CT (billions)	0.1	0.6	1.4	2.1	3.4
Neighbor Count (millions)	1.8	6.1	10.7	15.5	22.3
					
Compound Pairs per second	155,871	18,632	7,829	4,301	2,261
Conformer Pairs per second	155,871	146,318	156,241	156,618	153,959
Total Search Time (days)	8.2	68.5	163.1	296.9	564.9
Filtering Time	0.9%	0.8%	0.8%	0.7%	0.7%
Alignment Recycling Time	82.6%	86.0%	86.8%	86.8%	86.7%
Superposition Optimization Time	10.4%	10.0%	9.9%	10.2%	10.7%
Other Overhead Time	6.1%	3.2%	2.5%	2.3%	1.9%
Input data size (GB)	19	50	83	114	159

Performance of 3-D neighboring as a function of diverse conformer count for the 4,218 chemical structures (*Query*) with known biological function when neighbored against the entire "live" PubChem3D contents (*Search*) of 26,157,365 small molecules.

Alignment recycling is the next stage after filtering. This methodology consists of: comparing a shape fingerprint; locating common reference shapes; and then reuse of the alignment to the common reference, where the shape overlap and the feature overlap are computed at that recycled alignment to the reference shape. This is repeated for each common reference shape and only the best superposition is kept.

Alignment recycling provides two opportunities to further remove conformer pairs from consideration. If a reference shape cannot be found in common, the conformers are considered to be too different to be a neighbor. This alignment recycling fingerprint filter removes an additional 4% of all conformer pairs (14% of all conformer pairs not already filtered). If the pre-optimized best overlap from alignment recycling is not sufficiently large (yielding an ST of at least 0.735), the conformer pair is considered to be incapable of being a neighbor. This alignment recycling overlap filter removes 27% of all conformer pairs (96% of all conformer pairs not already filtered) but consumes 86% of CPU time. Together, all filtering steps remove 99.8% of conformer pairs prior to optimization of the conformer superposition at the recycled alignment. The final shape optimization step consumes 10% of the CPU time, retaining less than 0.6% of optimized conformer pairs as neighbor pairs. About 66% of conformer pairs shape-optimized are rejected due to an insufficient ST value (<0.795) to become a neighbor and the remainder rejected due to insufficient CT value (<0.495) at the shape-optimized superposition.

The overall throughput of the 3-D neighboring methodology is consistent across the range of diverse conformers considered, at a rate of ~150,000 conformers per second. The other overhead reported in Table 3 involves mostly the billions and trillions of timing measurements but also involves some memory allocation aspects. In reality, with timing statistics turned off, there is very little other overhead to the method. While the total size of the input binary data files grows as a function diverse conformer count, ranging from 19 GB to 159 GB, the computational density is more than sufficient to avoid making input of these search files a bottleneck, provided at least four conformers are being queried simultaneously. If fewer than four conformers are queried at a time, and the input binary files are not memory resident, input can be a bottleneck.

### 6. Alignment recycling

**Figure 11:**
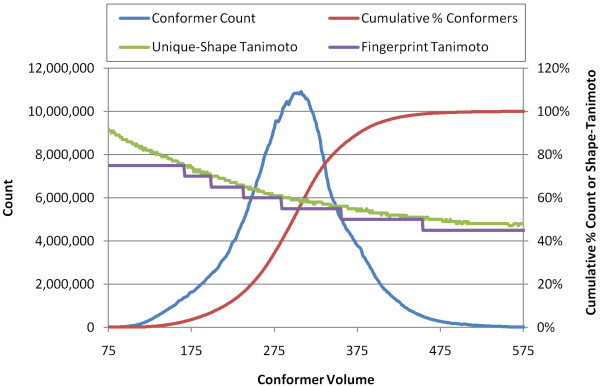
**Shape fingerprint design**. Plot of conformer count (blue line) [left Y-axis], cumulative % conformers (red line) [right Y-axis], unique-shape Tanimoto (green line) [right Y-axis], and fingerprint Tanimoto (purple line) [right Y-axis] as a function of conformer volume (Å^3^). The fingerprint Tanimoto indicates the seven volume regions of the reference shape and its corresponding ST minimum distance between reference shapes.

The alignment recycling methodology [14] was extended to cover non-hydrogen atom counts from 0-50 and rotatable bond counts from 0-15. This was achieved by leveraging our recent study on the diversity of shape space [15], where shape space was shown to grow gradually as a function of conformer volume and a dynamic shape similarity threshold for a relatively constant count of reference shapes. This curve (the Unique-Shape Tanimoto in Figure 11) was used to effectively partition shape space into seven regions. Each fingerprint region has a distinct shape similarity threshold (the Fingerprint Tanimoto in Figure 11) and covers the entire shape diversity of a given conformer volume range. As Table 4 shows, there are a total of 3,311 reference shapes across all seven regions, representing the entire shape diversity of 5.2 billion conformers for the entire contents (live and non-live) of the PubChem3D system (+45.9 million small molecules).

**Table 4:** Shape fingerprint design.

Volume range (Å^3^)	Fingerprint ST ()	Unique shape counts	Conformer count (millions)
1-165	0.75	335	56.2
166-199	0.70	341	133.6
200-238	0.65	307	415.7
239-285	0.60	393	1,155.7
286-344	0.55	408	2,210.5
345-432	0.50	559	1,150.6
433-999	0.45	968	114.8

**Total**		**3,311**	**5,237.1**

Fingerprint Tanimoto (), unique shape counts in the shape fingerprints, and the conformer counts spanned by the fingerprints.

**Figure 12:**
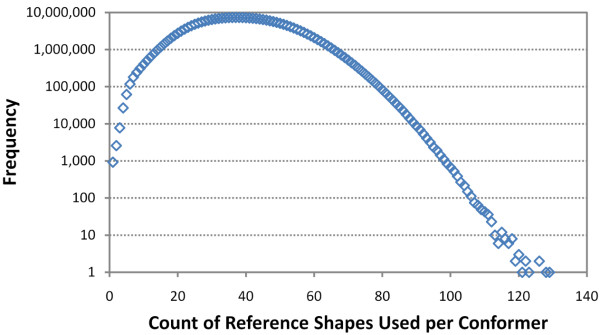
**Shape fingerprint bits are sparsely set**. Frequency plot of the total count of fingerprint reference shapes set per conformer for the first ten conformers of the 26,157,365 PubChem3D Compound records in the *Search *set, corresponding to 246,874,949 conformers.

**Figure 13:**
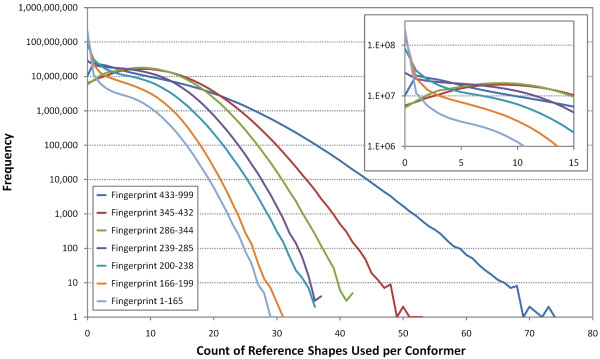
**Some shape fingerprint volume regions are mostly unused**. Plot of the frequency of the shape fingerprint bit counts per fingerprint volume region for the first ten conformers of the 26,157,365 PubChem3D Compound records in the *Search *set, corresponding to 246,874,949 conformers. A significant percentage of the conformers do not use fingerprint reference shapes in the volume ranges 1-165, 166-199, and 200-238.

When computing the shape fingerprint of a conformer, if a reference shape has a shape optimized superposition that is greater than or equal to the fingerprint shape similarity threshold (), the corresponding 3-D fingerprint bit is set. Although there are 3,311 reference shapes, the reference shapes utilized per conformer is relatively few. As shown in Figure 12, for the first ten diverse conformers from the 26.1 million compounds (246 million conformers) covered in the study of 4,218 small molecules of biomedical interest, there are at most a total of 129 reference shapes used per conformer, with an average and standard deviation of 39 +/- 13. This sparseness is to be expected as the shape fingerprint primarily identifies a specific region of shape space. Figure 13 depicts the count of set bits per fingerprint region across the 246 million conformers. As Table 4 shows, each fingerprint area covers a specific volume range. So, one should not expect a conformer with volume 100 Å^3 ^to have reference shapes in the conformer volume range 433-999, and vice versa. In fact, while each conformer has at least one reference shape set, many of the 246 million conformers considered do not have any reference shapes set in one of the seven different fingerprint regions. For the fingerprint reference shape volume (Å^3^) ranges 1-165, 166-199, 200-238, 239-285, 286-344, 345-432, and 433-999, a total of 83.2%, 62.4%, 35.1%, 11.6%, 2.4%, 2.6%, and 4.1% of the 246 million conformers, respectively, are not using the fingerprint region. This is reflected in the relatively high counts of conformers with no reference shapes, as depicted in the magnified section of Figure 13.

**Figure 14:**
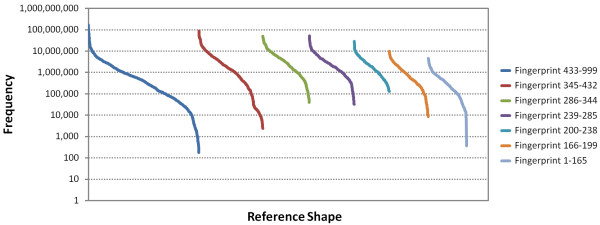
**Frequency of fingerprint reference shape use**. The frequency of use of the 3,311 fingerprint reference shape bits, separated by fingerprint volume region, by the first ten conformers of the 26,157,365 PubChem3D Compound records in the *Search *set, corresponding to 246,874,949 conformers. Some reference shapes are very popular while others are rarely used.

The relative popularity of each discrete 3,311 reference shapes varies markedly. Depicted in Figure 14, one can see the frequency of use of each reference shape defined in a given fingerprint volume range for the 246 million conformers. In each fingerprint volume range, there exist a very small number of reference shapes that clearly stand out as being used most often. Afterwards, the use of individual reference shapes falls off sharply and then gradually, until only peripheral reference shapes that are rarely used are left. This motif is seen for all fingerprint volume regions and may reflect the relative uniqueness (or lack thereof) of shapes across the first ten diverse conformers in PubChem.

### 7. Superposition storage

Superposition of two conformers requires modification of the coordinates of one conformer relative to the other. Retention of the rotational matrix and translation vector is a practical approach to retain a superposition between conformers to avoid having to re-compute a superposition or store modified coordinates of a conformer.

Storage of superposition results in PubChem3D involves identification of: the two conformers involved, often with one of the two conformers implicitly identified (*e.g*., by storing the superposition as a subordinate property of a conformer); the 3 × 3 rotation matrix; and the 3 × 1 translation vector. The PubChem3D conformer ID is often represented as either a 64-bit unsigned integer (sometimes stored in 16-character hex form), with the 32-high bits representing the PubChem Compound identifier (CID) and the 16-low bits representing the local conformer ID (LID), or two numbers "." separated (e.g., CID.LID). Storage of the rotation and translation parts represents more of a challenge, given there are twelve floating point numbers to convey. To provide for a more compact superposition representation, the ability to pack/unpack the rotation and translation into a 64-bit unsigned integer was developed. While described in more detail in the **Materials and Methods **section below, this involves transforming the rotation matrix into a quaternion and packing each of the four (Q_w_, Q_x_, Q_y_, Q_z_) components into 32-bits, 8 bits each. The remaining 32-bits are used to encode the translation vector.

**Figure 15:**
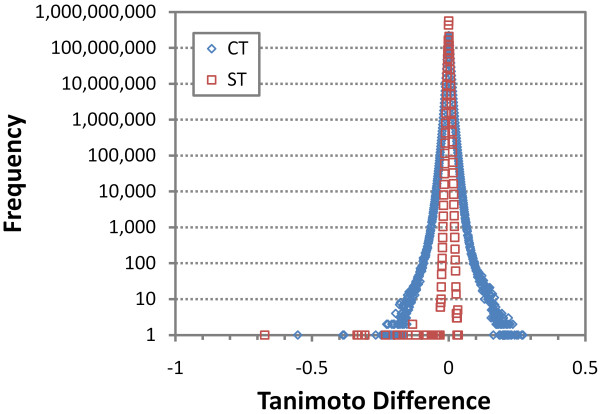
**Effect of superposition packing on ST/CT**. The difference in the ST/CT scores (binned in 0.001 increments) before and after packing superposition translation/rotation information into an unsigned 64-bit integer. A positve difference indicates an enhancement of the ST/CT scores and a negative difference indicates an error in the ST/CT scores.

To study the loss in accuracy due to encoding/decoding the conformer superposition information into a 64-bit integer, 1.85 billion unique conformer neighbor pairs in the 0-20 million CID range involving conformers that are the first diverse conformer of a compound were used. The chemical structure and 3-D coordinates of each conformer pair were: downloaded from the PubChem3D data system database; the superposition between the conformers was optimized, yielding a before ST/CT value pair; the superposition rotation and translation was encoded, decoded, and applied to the original downloaded conformer pair coordinates; and then a single point ST and CT value was computed, yielding an after ST/CT value pair. The difference in the before/after ST and CT values were binned in 0.001 increments and the population of the occupied bins are plotted in Figure 15 and summarized in Table 5.

**Table 5:** Effect of superposition packing on ST/CT.

Matrix Encoding Error Count			Matrix Encoding Enhancement Count		
	
Threshold	Count	Probability	Threshold	Count	Probability
<-0.01 ST	1,688,710 (0.09%)	1 in 1,096	>+0.01 ST	1,806,096 (0.10%)	1 in 1,024
<-0.025 ST	127 (0.00%)	14,566,929	>+0.025 ST	54 (0.00%)	34,259,259
<-0.05 ST	19 (0.00%)	97,368,421	>+0.05 ST	0 (0.00%)	-
<-0.10 ST	13 (0.00%)	142,307,692	>+0.10 ST	0 (0.00%)	-
					
<-0.01 CT	48,783,505 (2.64%)	1 in 38	>+0.01 CT	61,901,046 (3.35%)	1 in 30
<-0.025 CT	1,937,802 (0.10%)	955	>+0.025 CT	2,661,967 (0.14%)	695
<-0.05 CT	48,485 (0.00%)	38,156	>+0.05 CT	55,429 (0.00%)	33,376
<-0.10 CT	1,433 (0.00%)	1,290,998	>+0.10 CT	1,706 (0.00%)	1,084,408
<-0.15 CT	172 (0.00%)	10,755,814	>+0.15 CT	237 (0.00%)	7,805,907

Effect of encoding and decoding the rotation matrix and translation vector on the ST and CT values into a 64-bit unsigned integer across 1.85 billion 3-D neighbor conformer pairs.

Perhaps most remarkable, the superposition encode/decode procedure is just as likely to enhance the ST and CT values as detract from them. Also interesting is that the CT error curves are much broader, reflecting, in part, the much greater positional sensitivity of the CT measure. Small deviations in rotation have an increasing effect the further an atom is from the molecule center. Fictitious feature atoms are relatively sparse, have small atomic radii, and are often close to the periphery of the chemical structure. Shape similarity, on the other hand, is not as sensitive, as real atoms are relatively dense and most atoms in the molecule are typically near the steric center, thus, fewer atoms are affected from rotation encoding effects. As a whole, the use of a 64-bit integer to store a conformer pair superposition results in relatively few cases where the Tanimoto difference (after-before) is less than 0.025, with the chances for this to occur for ST and CT being 1 in 14.6 million and 1 in 955, respectively. If the error from being off a small fraction of a degree from the original superposition is too much, one could simply re-optimize the conformer superposition provided by PubChem, as the benefits in terms of the ease of storage are considerable.

## Conclusion

In the present paper, the PubChem 3-D "Similar Conformers" neighboring relationship and the methodology used in its computation are described. PubChem 3-D neighbors are defined as any two conformers with a shape-optimized superposition yielding a similar 3-D conformer shape (ST value of ≥ 0.8) and similar 3-D orientation of functional groups typically used to define pharmacophore features (CT value of ≥ 0.5). In the cases of chemical structures without features, a similar 3-D conformer shape with ST value of ≥ 0.93 is used.

To make the calculation of this 3-D neighboring relationship tractable, a series of filters were designed to avoid the time-consuming shape-superposition computation between conformer pairs that could not possibly be 3-D neighbors. This resulted in an average throughput of 150,000 conformer pairs per second per processor core, a speed sufficient to consider multiple diverse conformers per compound in the 3-D neighboring relationship.

Neighboring the first two diverse conformers of 26.1 million PubChem Compound records yielded 8.16 billion 3-D conformer neighbor pairs and 6.62 billion 3-D compound neighbor pairs, with an average of 253 "Similar Conformers" per PubChem Compound. Comparison of the PubChem 3-D "Similar Conformers" neighboring relationship with the PubChem 2-D "Similar Compounds" neighboring relationship using three well-known bioactive molecules (aspirin, caffeine, and morphine) showed a considerable degree of uniqueness between the two neighboring relationships and providing a number of related structures with significant biological annotation. This was also illustrated by the ability of the 3-D neighboring relationship to associate eight selected non-steroidal anti-inflammatory drugs (NSAIDs) to each other, despite little 2-D pair-wise similarity between most of the compound pairs. Additional study of 4,218 small molecules of biomedical interest across a range of diverse conformers shows that neighboring more conformers per compound will result in being able to associate more chemical structures to each other; however, an exponential increase in the count of conformer pairs considered results in only a linear increase in additional compound 3-D neighbor pairs.

## Materials and methods

### 1. Chemical structure 3-D representation

Theoretical 3-D descriptions of the 26,157,365 chemical structures covered in this work and found in the PubChem Compound database [1,2] are generated as described in our previous studies [15,28]. It is important to note that these conformers are not energy minima on a potential energy surface, but an ensemble of energetically-accessible (at room temperature), biologically-relevant (able to reproduce most known bioactive conformations), sampled (with a minimum atom pair-wise RMSD separation) conformations that the molecule may cover. In theory, these ensembles describe all relevant molecular shapes (including all important energy minima) within the resolution of the clustering RMSD for the conformer ensemble.

### 2. Molecular shape and features

An atom-centered Gaussian description [9-12] using Bondi radii [29] is utilized to compute 3-D similarity. Fictitious "feature" atoms (also known as "color" atoms) are defined to represent the pharmacophore feature functional group types present in a chemical structure. The Mills/Deans implicit force-field [30], as implemented in the OEShape C++ Toolkit, is employed to identify these features. The six feature types recognized are: anion, cation, hydrogen-bond donor, hydrogen-bond acceptor, hydrophobe, and ring. Feature atom 3-D coordinates are computed relative to the steric center of real "parent" atoms that comprise each feature. Post processing of feature atom assignment identifies any features of the same type within 1.0 Å of each other and merges the unique parent atoms that comprise the two features. This post processing step is performed iteratively, until no additional features are merged. The radius used for all feature atoms is 1.08265 Å.

Shape similarity computation utilizes the shape Tanimoto (ST) via Eq. (2) and only considers the non-hydrogen atoms in the molecule. Feature similarity, unlike shape similarity, involves summing the individual overlaps of the six component feature atom types when computing the *A*, *B*, and *AB *found in Eq. (2); thus, yielding Eq. (3) for the feature similarity measure, color Tanimoto (CT). Otherwise, the feature similarity computation method is identical to the shape similarity computation method.

When optimizing the shape superposition between a conformer pair, the OpenEye OEShape C++ toolkit is used via the OEBestOverlay object, with the parameters OEOverlapRadii::All and OEOverlapMethod::Analytic. Any other shape or feature computation utilizes in-house implementations using the Grant and Pickup [9] Gaussian-based shape methodology. For all in-house shape-based approaches, an exponent lookup table of size 6,001 is used in lieu of exponent calculation for the range of (-12.0 to 0.0) in 0.002 increments. Exponent values outside of this range are zero. All other terms in the Grant and Pickup shape-based methodology are computed exactly.

A grid-based approach is used by parts of the 3-D neighboring methodology to estimate the shape overlap with O(N) computational complexity. In these cases, a 3-D lattice of points separated by 0.25 Å give the shape overlap of a carbon probe-atom at the grid point to the query conformer. A cut-off distance of 4.5 Å is used for each query conformer atom, where no additional contribution to shape overlap is considered.

### 3. Diverse conformer concept

Although the theoretical conformer ensemble for each molecule may have up to 500 conformers (averaging ~110), it is not practical to consider all conformers for PubChem 3-D neighboring. Therefore, a diverse conformer concept is introduced that orders the conformers in the ensemble for a chemical structure by their combined shape and feature dissimilarity, with the most dissimilar conformers first. The lowest-energy conformer in the conformer ensemble is selected as the default, first diverse conformer to seed the process. The conformer with the least combo Tanimoto (being the sum of the ST and CT similarity values for the ST-optimized superposition) to the first conformer is selected as the second most diverse conformer. The conformer with the least sum of combo Tanimoto to the first two conformers is selected as the third, and so on until all conformers are assigned a diverse conformer ordering. In the case of a tie, the conformer with the largest sum of combo Tanimoto to all unassigned conformers is selected. If a tie persists, the conformer with the least LID (local conformer identifier) is selected.

### 4. Shape fingerprint definition

Haigh *et al*. [13] applied a clustering technique to select a diverse set of reference shapes that cover the overall shape space of possible 3-D shapes, and generated 3-D molecular shape fingerprints using these reference shapes. Comparison of molecular shape fingerprints was shown to be orders of magnitude faster than shape-overlap-based approaches such as ROCS [10], illustrating its potential in screening a large 3-D chemical database. Therefore, we applied the 3-D shape fingerprint technique, in conjunction with "alignment recycling" [14], for use in computing a "Similar Conformer" relationship.

In our recent study [15], a dynamic shape similarity threshold (*ST^thresh^*) was employed in clustering conformers of a particular volume such that the resulting reference shape count became less than or equal to a certain number (200). In this manner, the number of reference shapes per volume can be kept relatively constant while the growth of the shape space as a function of volume is manifest by a decrease in  (the Unique-Shape Tanimoto in Figure 11). The plot of the Unique-Shape Tanimoto versus the conformer volume was used to choose appropriate  values for the 3-D shape fingerprint generation (the Fingerprint Tanimoto in Figure 11). The  value was chosen to gradually decrease from 0.75 to 0.45 (with a decrement of 0.05) as the conformer volume increases, resulting in seven different regions of conformer volume according to their  values. The reference shapes of each region obtained from the previous study [15] were then pooled and clustered at . Following this step, all conformers in PubChem3D within the given volume range region were examined to locate any additional reference shapes. Also, as new chemical structures are added to PubChem, they are examined to identify new candidate reference shapes.

The resulting "unique shape" count is listed in Table 4, with the conformer count that belongs to the shape space represented by the corresponding unique shapes. It indicates that the shape space spanned by 5.24 billion conformers (the entire contents of the PubChem3D archive, live and non-live, from more than 45.9 million unique chemical structures) can be represented in such a manner so as to only require 3,311 unique reference shapes (a number which may grow as a function of time). Figure 14 shows the frequency of use of the various 3-D fingerprint reference shapes, with some being heavily utilized while others are rarely used. The volume range 433-999 is the largest in both volume spanned and count of reference shapes. We anticipate that this volume range may need to be split into separate regions in the future.

### 5. PubChem 3-D neighbor processing

PubChem Compound (CID) records are partitioned into two sets, "live" and "non-live". A "live" CID is one that has at least one current version PubChem Substance record pointing to it. The "non-live" partition contains all CIDs not considered to be "live". For each record that is contained in the PubChem3D system and considered to be "live", PubChem computes a "Similar Conformer" relationship that considers both shape similarity (ST ≥ 0.8) and feature similarity (CT ≥ 0.5). [Chemical structures without features, while rare, can have a similar conformer relationship with other featureless chemical structures provided the ST ≥ 0.93.] Essentially, this amounts to a 3-D similarity search of a given conformer across the first N-diverse conformers of "live" CIDs, where at the time of writing "N" is two, with a third in the process of being added. This processing we call "neighboring".

This PubChem3D similarity search is a multistep process designed: to filter out conformer pairs that cannot possibly be neighbors, to generate a near-optimal superposition between conformer pairs, and to perform a final optimization of the superposition to maximize the shape volume overlap between conformer pairs. These distinct stages are described below.

#### Stage 1: Filtering

There are multiple filters used in PubChem3D neighboring, each with different degrees of computational cost and efficiency. The cheapest filters utilize the ST and CT equations [Eq. (2) and Eq. (3)], the pre-computed self-overlap volumes (*A *and *B*), the predefined Tanimoto thresholds, and a rearranged Tanimoto equation solving for *AB*, which now represents the minimum overlap (*AB_min_*) necessary to meet the threshold criteria (ST or CT). If this *AB_min _*is greater than either *A *or *B*, then the conformer pair may be eliminated from further consideration as the maximum possible overlap between the conformer pair will be smaller than *AB_min_*, yielding a Tanimoto value less than the respective threshold. The resulting equation for CT, using the threshold of 0.5, is:(4)

This approach to filtering can be used a second time (although at greater computational cost) for feature similarity by using the individual feature counts. For each feature type, one uses the feature count (*A *and *B*) and the common count (either *A *or *B*, whichever is the lesser). One can then compute a *CT_max _*that must be above the threshold of 0.5 to possibly be a neighbor, as shown in Eq. (5):(5)

An enormous advantage to this filter is that it operates on compound pairs. This means it applies to all conformers being neighbored for the respective compound pair, unlike the previous CT filter which is per conformer pair. So, as the count of conformers per compound is increased, the utility of this filter is magnified.

Taking the exact same approach for ST as CT is not possible, as the shape volume *AB *overlap can be greater than either *A *or *B*, due to the Grant and Pickup atom-centered Gaussian-based formulation of molecular shape, the radii used, and the bond distances of atoms. The net effect is such that the *AB *overlap can be significantly greater than either *A *or *B*. The filter for ST (and the threshold of 0.8) becomes:(6)(7)

The ratios in both Eqs. (6) and (7) must be between 0.75 and 1.50 for the conformer pair to possibly be a neighbor.

#### Stage 2: Shape fingerprint comparison and alignment recycling

The next stage of filtering utilizes the PubChem3D shape fingerprint computed for each conformer. For a given conformer pair to become neighbors to each other, there must be a common reference. If a common reference cannot be found, the conformer pair cannot be neighbors and no further computation is necessary. For each common reference, the 3 × 3 rotation matrix and 3 × 1 translation vector that aligns the conformer to the reference is utilized. The resulting 3 × 3 rotation matrix and 3 × 1 translation vector are applied to the coordinates of one of the conformers. This provides a superposition of one conformer to the other. A combined shape and feature overlap for the conformer pair using this alignment is then computed as following:(8)

where *AB_shape _*is the common shape volume between the conformer pair (using a precomputed shape-grid at 0.25 Å resolution) and *AB_feature _*is the sum of six component feature overlaps (using a feature atom radius of 1.25 Å, to account for proximate color atoms).

This approach is repeated for each common reference shape. The reference-shape-derived conformer alignment yielding the largest *AB_combo _*value is used in a final *AB_shape _*overlap computation, this time not using a grid-based approach. The final *AB_shape _*overlap is used along with the pre-computed self shape overlap values per conformer to compute the ST at this overlap geometry. If the computed ST is not greater than 0.735, the conformer pair is considered to not possibly be a neighbor. [The "grid" *AB_shape _*can be sufficiently different than the "exact" *AB_shape _*value, resulting in the loss of many neighbor relationships.]

#### Stage 3: Shape overlay optimization and ST/CT score computation

Using the "alignment recycling" conformer pair superposition from the previous stage, a final superposition optimization to maximize the shape volume overlap between the conformer pair is performed using the OEShape C++ toolkit [12]. If the final conformer superposition yields an ST ≥ 0.8 (actually 0.795 after rounding to the nearest 0.01 is considered), the CT is also computed at the same conformer alignment. If CT ≥ 0.5 (actually 0.495 after rounding to the nearest 0.01 is considered), the conformer pair is considered to be a neighbor. As mentioned previously, if both conformers are devoid of features, alternatively, an ST ≥ 0.93 (actually 0.925 after rounding to the nearest 0.01 is considered) is sufficient to be considered a neighbor. The 3 × 3 rotation matrix and 3 × 1 translation vector and the ST/CT similarity values are retained for the conformer pair.

### 6. PubChem 3-D neighbor processing addendum

There are additional aspects to neighbor processing that are germane to their accuracy and use. To minimize input overhead and memory utilization, all input data is encoded in a highly compact 64-bit aligned binary format (gzip or bzip2 compression reduces file size by only 4%) that contains all information necessary to perform the neighboring computation. One side effect of this encoding is that conformer coordinates are transformed into an integer value with a resolution of +/- 0.0015 Å and restricts coordinates to the range (-50 Å, +50 Å), which is more than sufficient for all conformers in the PubChem3D data system. Another side effect of this encoding scheme is that, to obtain the superposition alignment between neighbored conformers, one must first transform the conformers to their steric center (*i.e*., subtract the coordinate average per axis) prior to applying the stored 3 × 3 rotation matrix and then the 3 × 1 translation vector (in that order). For conformers stored in the PubChem3D system, conformers are already at the steric center (and rotated into the non-mass weighed inertial frame of reference).

Another major consideration is that the 3 × 3 rotation matrix and 3 × 1 translation vector for all fingerprints and neighbor pairs are encoded and stored as a 64-bit unsigned number. This involves transforming the 3 × 3 rotation matrix into the quaternion and then encoding these four values (Q_w_, Q_x_, Q_y_, and Q_z_) and the 3 × 1 translation vector. Each quaternion value is encoded in eight bits with a range of (-1, +1) and a resolution of 0.00784. Care must be taken when either encoding or decoding these quaternion values to always normalize, as doing so reduces the encoding error. Encoding the 3 × 1 translation vector uses a slightly different approach to achieve a maximum range of (-100 Å, +100 Å). Three of the remaining 32 bits are used to hold the sign of the X, Y, and Z translation. The remaining 29 bits encode the three absolute values in the following fashion. A value of 1.0 is added and the log of the resulting number is taken and divided by 812 (812^3 = 535387328, which just fits within 29 bits) and rounded to the nearest whole number. The integer encoded X, Y, and Z are then combined as such:(9)

The result of using a log value provides for a translation encoding that gives the best precision at 0 Å (+/- 0.0028 Å) and the worst at 100 Å (+/- 0.29 Å). Considering the requirement that a conformer pair must be at the steric center prior to encoding, the encoding error due to translation is minimal.

Validation of the encoding/decoding accuracy as a function of ST and CT values across +1.85 billion unique neighbor pairs show (Table 5) that there is nearly an equal possibility to enhance the ST or CT value as it is to detract from it. However, the CT is much more sensitive to encoding/decoding than ST, with a 1 in 38 chance of yielding a value less by 0.01 than that reported. This is to be expected, as the radius used for feature atoms in CT computations is nearly 60% smaller than for carbon atoms, meaning small changes in the alignment can have a big effect on similarity, especially for features that are furthest from the steric center (a "torque arm" effect). Additionally, the shape overlap optimization does not consider feature atoms, meaning a small change in rotation or translation may either increase or decrease the CT value, considering no attempt was made to optimize feature alignment.

## Competing interests

The authors declare that they have no competing interests.

## Authors' contributions

EEB performed most of the research and SK wrote the first draft. SHB reviewed the final manuscript. All authors read and approved the final manuscript.

## Supplementary Material

Additional file 1 **List of PubChem Compound identifiers (CIDs) that were neighbored using different counts of diverse conformers per compound**. Click here for file
